# When Immunotherapy Hits the Joints: A Case of Late-Onset Polyarthritis Induced by an Immune Checkpoint Blockade and Literature Review

**DOI:** 10.7759/cureus.106543

**Published:** 2026-04-06

**Authors:** Yuenu Wu, Alexander Digregorio

**Affiliations:** 1 Internal Medicine, Phoenixville Hospital, Phoenixville, USA

**Keywords:** arthritis, immune checkpoint inhibitor, late onset immune-related adverse event, nivolumab, prostate cancer

## Abstract

Immune checkpoint inhibitors (ICPis) are increasingly used across a wide range of malignancies, underscoring the growing need for awareness of ICPi-related adverse events (irAEs), especially outside cancer centers. We report a case of polyarthritis developing over 15 months after the initiation of nivolumab in a patient with advanced urothelial carcinoma of the prostate. The patient presented with symmetrical polyarthritis characterized by inflammatory features, which responded well to corticosteroid therapy. Given the delayed onset of symptoms following ICPi initiation, an extensive infectious and autoimmune workup was conducted, all of which returned negative. This case underscores the importance of continued vigilance for irAEs even during long-term therapy. Timely recognition of ICPi-induced arthritis is crucial, as early initiation of corticosteroids can lead to rapid symptom relief and significant improvement in quality of life.

## Introduction

Immune checkpoint inhibitors (ICPis) have emerged as a standard therapeutic approach for various advanced malignancies; however, they are also linked to a range of irAEs that can impact nearly any organ system [1,2]. Nivolumab (Opdivo, Bristol-Myers Squibb, Princeton, New Jersey, US), a monoclonal antibody that blocks the interaction between a protein receptor programmed cell death 1(PD-1) and its ligands, PD-L1/PD-L2, restores T-cell activity within the tumor microenvironment by inhibiting the immune checkpoint signaling that normally inactivates T-cells [3]. However, it can also lower immune tolerance to non-tumor tissues, potentially triggering inflammatory syndromes. It is important to acknowledge that the diagnosis may be delayed due to the variability in its presentation and onset. ICPi-induced polyarthritis is a relatively rare type of irAE, and reports of late-onset cases associated with nivolumab use are very limited.

We herein present a case of grade 3 nivolumab-induced polyarthritis with an atypically delayed onset, manifesting 60 weeks after treatment initiation, in contrast to the reported median onset of approximately eight weeks. Delayed presentations such as this may contribute to diagnostic challenges and may demand a multidisciplinary approach. Additional case reports are needed to better characterize the clinical patterns of delayed ICPi-induced arthritis and to facilitate earlier recognition and management.

## Case presentation

The patient is a 66-year-old Caucasian male with a history of advanced prostate cancer and ADHD who presented to the emergency department with severe knee pain and swelling. Given his systemic inflammatory response syndrome (SIRS)-like presentation with tachycardia and leukocytosis, he was admitted to the inpatient medicine service for further evaluation. His oncologic history is significant for metastatic prostate adenocarcinoma with bone involvement and urothelial carcinoma affecting overlapping regions of the bladder and prostate. He was initiated on degarelix (Firmagon) and denosumab (Xgeva) for prostate cancer management, completed a gemcitabine plus cisplatin (Gem/Cis) regimen, and underwent laparoscopic robotic radical cystoprostatectomy with urinary diversion (ileal conduit and neobladder) and bilateral pelvic lymph node dissection in October 2023. Pathology demonstrated ypT0 ypN1 (1 positive lymph node), cM0. Given the node-positive residual urothelial carcinoma, adjuvant nivolumab (480 mg every 4 weeks) was initiated in November 2023. Treatment was briefly suspended for one week (40 weeks after initiation) due to colitis, which resolved promptly with a short course of prednisone (Table 1), allowing nivolumab to be resumed without further complications.

**Table 1:** Summarized irAEs irAEs: immune checkpoint inhibitor-related adverse events

irAEs	Onset	Rx	Clinical response
Dermatitis, grade 1	12 weeks	Topical betamethasone	Remission
Colitis, grade 2	40 weeks	Prednisone 40 mg taper. Nivolumab was suspended for 1 week	Remission
Thyroiditis, grade 2	60 weeks	Synthroid 50 mg daily	Stable
Polyarthritis, grade 3	60 weeks	- Prednisone 1 mg/kg taper - HCQ 400 mg daily - MXT 25 mg qw - Discontinuation of Nivolumab	Stable

After 60 weeks of therapy, the patient developed progressively worsening, symmetrical joint pain and swelling involving the shoulders, elbows, knees, and both metacarpophalangeal (MCP) and proximal interphalangeal (PIP) joints, with particularly prominent involvement of both knees. He reports morning stiffness lasting over 30 minutes, which has significantly impaired his daily functioning, including ambulation and small tasks such as opening containers. He denies fever, skin rashes, ocular ulcerations, sicca, or muscle pain or weakness. Physical examination revealed diffuse joint swelling and tenderness without associated erythema or warmth. Diagnostic arthrocentesis of the knee demonstrated inflammatory synovial fluid changes (Table 2), with no crystal deposits and no growth on microbiological cultures, Lyme PCR negative (endemic in the local region). Blood cultures were similarly negative, and serum procalcitonin levels were within normal limits. Serologic testing for parvovirus IgM (0.2, Index/ratio, <0.9: negative), antinuclear antibodies (ANA), rheumatoid factor (RF, 8.4 IU/mL), and anti-cyclic citrullinated peptide (anti-CCP, <0.5 IU/mL) antibodies were negative. Radiographic imaging of the knees revealed no evidence of erosive changes or periarticular osteopenia. Laboratory findings demonstrated markers of systemic inflammation, including an elevated C-reactive protein (CRP) of 22.86 mg/L, erythrocyte sedimentation rate (ESR) of 73 mm/hr, ferritin level of 1107 ng/mL, and platelet counts ranging from 700 to 900 ×10³/µL. Based on the clinical presentation, lab and imaging findings, a diagnosis of grade 3 immune checkpoint inhibitor-induced arthritis was made. As we do not have an in-house rheumatologist for consultation, we started him on intravenous (IV) methylprednisolone at 1 mg/kg, followed the next day by an oral prednisone taper at 1 mg/kg over one month, per guideline recommendation [4,5]. Nivolumab was held. The patient experienced rapid symptomatic improvement and was discharged home with plans to follow up with a rheumatologist. Unfortunately, his symptoms worsened as the steroid dose was reduced. Hydroxychloroquine (HCQ) 400 mg daily was initiated by his rheumatologist, but it did not provide additional benefit.

**Table 2:** Study of synovial fluid

	Left knee	Right knee	Ref Range
Appearance	Yellow, cloudy	Yellow, cloudy	_
Crystals	Negative	Negative	Negative
Red blood cells (cells/uL)	2,000	4,000	0-2,000
Total nucleated cells (cells/uL)	15,952	6,085	0-20
Segmented cells (%)	94	93	0-50
Lymphocytes (%)	3	4	0-50
Monocytes (%)	3	3	0-50

**Figure 1:**
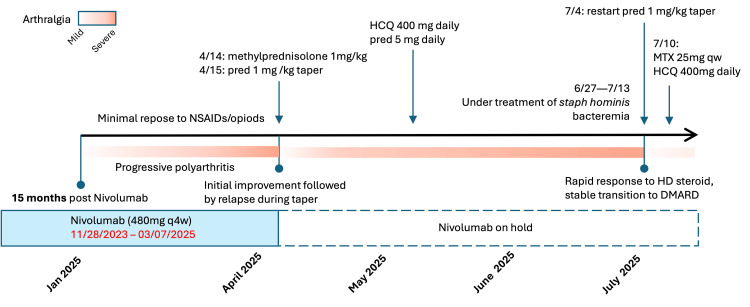
Timeline of clinical course Summary of the clinical course, including management of arthritis and treatment response, in relation to nivolumab therapy. MTX: methotrexate; HCQ: hydroxychloroquine; pred: prednisone; NSAIDs: nonsteroidal anti-inflammatory drugs; DMARD: disease-modifying anti-rheumatic drug; D/C: discontinue; HD: high dose

Two months after discharge, his clinical course was complicated by hospitalization for the management of *Staphylococcus hominis* bacteremia. Rheumatology was consulted due to significant polyarthritis while on HCQ 400 mg and prednisone 5 mg daily. Following infectious disease clearance, high-dose prednisone (1 mg/kg) with a four-week taper was restarted, resulting again in a rapid response. He continued close follow-up with rheumatology and was transitioned to disease-modifying antirheumatic drugs (DMARDs, methotrexate 25 mg weekly), achieving steady improvement in joint pain and overall functional status. Nivolumab was recommended to remain on hold indefinitely (Figure 1). A repeat prostate-specific membrane antigen (PSMA) PET/CT in June showed no new lesions, with mild improvement in the PSMA-avid left pelvic lymph node metastasis and bone disease. PSA trends also remained stable (6.02,7.87, 5.55, 4.7 ng/ml from April to July 2025) at the most recent follow-up.

## Discussion

This patient presented with delayed-onset ICPi-associated seronegative polyarthritis. He had no personal or family history of autoimmune diseases but a remote history of smoking, and remains in remission. A retrospective review of his clinical history reveals additional irAEs affecting the cutaneous, gastrointestinal, and endocrine systems (Table 1). Notably, he developed localized dermatitis of the lower back shortly after initiating nivolumab, which responded to topical betamethasone. Approximately 10 months into therapy, he experienced severe diarrhea, exceeding 10 bowel movements daily and significantly disrupting sleep, which resolved with a short course of oral prednisone. During the current admission, new-onset thyroid dysfunction was identified, with a markedly elevated thyroid-stimulating hormone (TSH) level of 11.344 mIU/L. It remains unclear whether patients who experience multiple early irAEs are at increased risk for subsequent late toxicities. Interestingly, Pozas et al. reported that most patients (92%) with late-onset irAEs had experienced an early-onset event [6]. Moreover, ICPi-induced arthritis is associated with increased odds of cutaneous, gastrointestinal, and endocrine irAEs [7,8].

**Figure 2:**
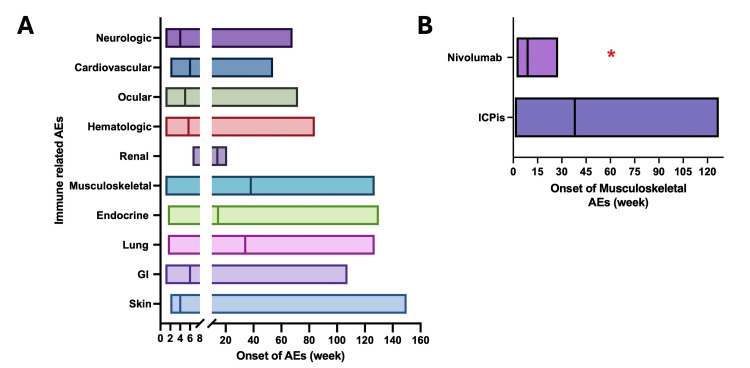
The median onset of common irAEs A: Onset of common irAEs. Graph using data from the ASCO guideline 2021 [4]. Boxes represent the reported range of onset times, and lines in boxes indicate the median time to onset. B: Comparison of onset timing between monotherapy nivolumab-related arthritis and general ICPi-associated arthritis, based on data (N=384) from Liu et al. [7], derived from the FDA Adverse Event Reporting System (FAERS) database, versus data (N=372) from a systematic literature review of 4339 articles by Ghosh et al. [22]. The red asterisk indicates the onset timing observed in our patient. GI: gastrointestinal; AEs: adverse events; iCPi: immune checkpoint inhibitors; irAEs: ICPi-related adverse events Graph generated using GraphPad Prism 10 (GraphPad, San Diego, California, US).

Immune checkpoints, including CTLA-4, PD-1/PD-L1, TIM3, LAG3, and TIGIT, function as physiological regulators that attenuate T-cell activation to maintain self-tolerance and prevent immune-mediated damage to normal tissues [3]. However, tumor cells frequently exploit these pathways to escape immune surveillance and elimination. ICPis have been developed to reinvigorate antitumor immunity by blocking these inhibitory signals, but they come with an increased risk of immune-related adverse events, as imbalanced T-cell activation with impaired regulatory T-cell function can disrupt peripheral tolerance and result in inflammatory damage to non-malignant tissues [3]. Of note, patients with pre-existing autoimmune conditions can also have flare-ups during ICPi therapy, but these conditions are not contraindicated for immune therapy [1,9,10]. The onset, frequency, and clinical manifestations of irAEs can vary depending on the patient’s intrinsic factors (gender, gut microbiome, cancer type, comorbidities), type/dose/duration of ICPi, and whether combination therapies are employed [1,7,11,12]. Most irAEs typically present within the first 8 to 12 weeks of therapy [4,13] (Figure 2); however, some manifestations, particularly those involving the musculoskeletal system, renal parenchyma, endocrine glands, and pulmonary tissue, may arise a little later, but often within the first 10 months of treatment (Figure 2). Late-onset irAEs are defined as immune-related adverse events that occur ≥12 months [6,14,15] after initiating immunotherapy or ≥3 months after its discontinuation [6]. These delayed presentations can pose diagnostic challenges, especially when symptoms overlap with disease progression or other comorbid conditions. Although less common than other irAEs [13], ICPi-induced arthritis occurs in approximately 0.81-7% [11,16] of patients, with the highest frequency and severity observed in those receiving combination ICPi therapy [11]. The median time of onset is approximately 16-19.2 weeks following ICPi initiation [17], and symptoms typically present early with nivolumab (Figure 2). The clinical spectrum is broad, ranging from mild arthralgia to monoarticular and oligoarticular arthritis, as well as polymyalgia rheumatica-like syndromes. Among these, polyarticular arthritis represents the most prevalent phenotype [12,17-20] and frequently mimics the clinical features of rheumatoid arthritis [16]. However, these cases are predominantly seronegative (negative for RF and anti-CCP antibodies) [16,21]. ANA positivity is usually comparable to that of the general population.

Most patients present with mild to moderate disease [12,17], with 36% and 49% experiencing grade 1 and grade 2 severity [22], respectively, while approximately 15% develop severe (grades 3-4) manifestations [22]. Mild presentation of arthritis can be managed with NSAIDs, and intra-articular corticosteroid injections can be considered for oligoarthritis. But starting grade 2 arthritis, where symptoms interfere with instrumental activities of daily living, systemic corticosteroids may be warranted and or combined with a temporary suspension of ICPi therapy. For more severe presentations--grade 3 and above--where there is a risk of permanent joint damage or severe functional impairment, escalation to synthetic or biologic DMARDs should be considered, along with possible referral to rheumatology [4]. Encouragingly, the majority of patients respond favorably to standard treatment approaches [22]. As such, timely recognition and intervention are critical to preventing joint erosions, preserving long-term function, and reducing the need for ICPi interruptions.

Research and reviews on irAEs continue to expand, accompanied by increasing standardization in diagnostic criteria, grading systems (Common Terminology Criteria for Adverse Events, CTCAE), and treatment approaches as recommended by current clinical guidelines [4]. Nevertheless, despite prevailing hypotheses [3,16,23] (aberrant activation of dormant pathogenic T-cell clones, dysregulated cytokine secretion/autoantibody production) implicating impaired immune tolerance, the pathophysiological mechanisms underlying irAEs remain poorly understood, particularly given their wide spectrum of clinical manifestations. The clinical significance of irAEs is also not fully understood. While some studies suggest a correlation between the occurrence of irAEs and improved disease control [18], progression-free survival (PFS) [21], and overall survival (OS) [13], these observations are often limited by small sample sizes, study heterogeneity, and retrospective nature. Otsuka et al. compared PFS and OS in patients with urothelial carcinoma treated with pembrolizumab and found improved outcomes in those who developed grade 2 irAEs compared to grade 0 or 1 [24]. However, this benefit was lost when irAEs progressed to grade 3 [24]. Notably, concerns remain regarding the use of immunosuppressants and withholding of ICPi to manage irAEs, particularly their potential impact on cancer progression. Although current evidence suggests a neutral effect on oncologic outcomes [1,17], ongoing research and future data are anticipated to provide greater clarity and revise management strategies.

As an internal medicine unit in a community hospital, when receiving patients with cancer undergoing immunotherapy who present with rheumatic or musculoskeletal symptoms, it is essential to differentiate non-rheumatic causes (infectious, inflammatory, or endocrine disorders), autoimmune conditions, tumor progression or infiltration, and paraneoplastic syndromes. In the case of our patient, the polyarthritis is not attributed to infectious etiology (bacterial or viral), inflammatory condition (osteoarthritis or crystals ), systemic autoimmune condition, or tumor progression (imaging study with CT chest/abdomen/pelvis and PSA level are stable). Additionally, paraneoplastic syndromes, such as palmar fasciitis and polyarthritis syndrome in advanced prostate cancer, are unlikely given the patient’s prompt and favorable response to corticosteroid therapy.

The limitation of this case report is that we were unable to perform more advanced imaging studies, such as MRI, at our community hospital. As we do not have an in-house rheumatologist, the patient continued follow-up at an outside clinic. His treatment course was unfortunately disrupted by the episode of *Staphylococcus hominis* bacteremia, which delayed the re-initiation of high-dose steroids after failure of low-dose prednisone and HCQ. Despite the unusual late presentation, his rheumatologist agreed with the diagnosis and initiated DMARD therapy to allow transition off steroids. Nivolumab was held indefinitely, given the severity of symptoms. In managing this patient, an initial four-week corticosteroid taper produced a marked early response but was followed by symptom recurrence [25]. This case suggests that a slower taper (>6 weeks) may be more effective [25], which could inform the management of other late-onset polyarthritis. However, further data are needed to evaluate the safety and efficacy of prolonged corticosteroid tapering in similar cases. Clinically, the patient experienced improvement in joint pain and functional status; however, no standardized assessment tools or serial laboratory markers were available to objectively quantify his response, as direct follow-up could not be performed.

Table 3 lists other cases of nivolumab-induced arthritis reviewed in this study.

**Table 3:** Nivolumab-induced arthritis irAEs: immune checkpoint inhibitor-related adverse events

irAEs	Onset/Median onset	Cancer type	Sample size	Reference
Arthritis, grade 1-2	3.3 month	Unspecified	17	Cappelli et al. [20]
Polyarthritis, grade 2	1 month	melanoma	1	Smith-Uffen et al. [26]
Arthritis, unspecified grade	10th infusion ( presumed 5-10 months)	Hodgkin’s lymphoma	1	Çolak et al. [27]
Polyarthritis, grade 3	5.5 month	Adenocarcinoma (lung vs. gastrointestinal)	1	Thapa et al. [28]
Polyarthritis, grade 3	3 days	melanoma	1	Cure et al. [29]
Arthritis, unspecified grade	~ 2 months (62 days)	Unspecified	384	Liu et al. [7]

## Conclusions

We aim to highlight the importance of recognizing late-onset irAEs for specialties outside Oncology and Rheumatology, particularly in light of the expanding use of ICPi therapies across various malignancies. Such cases are becoming more frequently encountered outside cancer centers. Although typically not life-threatening, ICPi-related polyarthritis can significantly impair quality of life and functional capacity. It is essential to maintain a high index of suspicion for this adverse event, even when symptoms arise outside the typical onset window, to ensure timely diagnosis and initiation of treatment that can substantially improve daily functioning. Standardized follow-up questionnaires can greatly assist the early detection of irAEs in the Oncology office setting, and should be shared in the electronic medical record (EMR), which can increase awareness in other specialty visits. Additionally, further research into the pathophysiology of immunodysregulation in late-onset ICPi-induced arthritis, along with studies assessing its clinical features and the safety and efficacy of prolonged corticosteroid tapering, is needed. Such work may facilitate earlier diagnosis and advance therapeutic strategies to improve patient outcomes.
